# Anti-Wrinkle Efficacy of Edible Bird’s Nest Extract: A Randomized, Double-Blind, Placebo-Controlled, Comparative Study

**DOI:** 10.3389/fphar.2022.843469

**Published:** 2022-03-09

**Authors:** Hyung Mook Kim, Yong Moon Lee, Ee Hwa Kim, Sang Won Eun, Hyun Kyung Sung, Heung Ko, Sang Jun Youn, Yong Choi, Wakana Yamada, Seon Mi Shin

**Affiliations:** ^1^ Global Cosmeceutical Center, Cheongju-si, South Korea; ^2^ College of Pharmacy, Chungbuk National University, Cheongju-si, South Korea; ^3^ Daehan Chemtech Co., Ltd., Seoul, South Korea; ^4^ Department of Pediatrics, College of Korean Medicine, Semyung University, Jecheon-si, South Korea; ^5^ Department of Internal Medicine, College of Korean Medicine, Semyung University, Jecheon-si, South Korea; ^6^ RnBS Corp., Seoul, South Korea; ^7^ New Products Development Department, Oryza Oil & Fat Chemical Co., Ltd., Ichinomiya, Japan

**Keywords:** bird nest extract, placebo-controlled study, randomized trial, skin health, nutritional supplements

## Abstract

This study aimed to evaluate skin health’s functional improvement, such as wrinkles, elasticity, moisture, and whitening, and safety following the consumption of “edible bird’s nest extract” for 12 weeks by women. This single-center, double-blinded, parallel-group, placebo-controlled study included women aged 40–60 years. Our primary purpose was to assess improvement in skin wrinkles, elasticity, and moisture after 12 weeks using an SV700, cutometer, and corneometer, respectively, compared to baseline measurements. Our secondary purpose was to evaluate skin wrinkle, elasticity, and moisture changes at 4 and 8 weeks from baseline using the aforementioned equipment, and measure transdermal water loss and melanin and erythema indexes using a tewameter and mexameter, respectively. Experts performed the visual evaluation of skin wrinkles at 4, 8, and 12 weeks from baseline. The participants were randomly allocated in a 1:1 ratio into the edible bird’s nest extract or the placebo group with 43 participants each, where they consumed 100 mg of the extract or placebo, respectively, daily for 12 weeks. The outcomes were measured at every visit. In this study, upon comparing changes in the skin elasticity value between the two intake groups at 12 weeks of ingestion, skin elasticity in the edible bird’s nest extract group decreased significantly compared with that in the placebo group. Adverse reactions were absent in both groups. In the case of laboratory test results, changes before and after the ingestion of the extract were within the normal range, thus indicating no clinically significant difference. The edible bird’s nest extract was effective in improving skin wrinkles. Moreover, it is beneficial for skin health and can be used as a skin nutritional supplement. Compared with the placebo, the edible bird’s nest extract was identified as safe.

**Clinical Trial Registration:** https://cris.nih.go.kr/cris/search/detailSearch.do?search_lang=E&search_page=M&pageSize=10&page=undefined&seq=21007&status=5&seq_group=20330, identifier KCT0006558.

## Introduction

Edible bird’s nest (EBN) is the swift’s nest that is made from its saliva and contains sialylglycoconjugates. The composition of the swift’s saliva resembles that of salivary mucin. Many studies have been carried out on the tonic effects of EBN, and it has been shown that EBN stimulates mitosis hormones and the epidermal growth factor, resulting in the repair of cells and stimulation of the immune system (Guo et al., 2017). In China, it is termed “Yan Woo (燕窩)” and was used to maintain the beauty and youth of court and upper-class ladies. In addition, according to the ancient Chinese literature, “Bencao gangmu shiyi (本草綱目拾遺) and Bencao qiu zhen (本草求眞),” it is effective in curing tuberculosis, chronic diarrhea, and lung infections (Dictionary of Chinese Traditional Drugs, 1985). According to Chinese medical books, it brightens the skin, nourishes the stomach and lungs, and improves complexion. Although previous studies showed that EBN contains sialic acid, is effective in brain development, and is used for the treatment of various chronic inflammatory diseases, studies on its effects on skin health are still lacking (Kim et al., 2021). Sialic acid is a generic term for acyl derivatives of neuraminic acid, and there are more than 30 kinds of this derivative in nature (Kiefel and von Itzstein, 2002). N-acetylneuraminic acid is present in the EBN extract, which improves learning ability (Morgan and Winick, 1980), strengthens immunity (Bagriaçik and Miller, 1999), prevents influenza infection (Biddle and Belyavin, 1963), and acts as an important ingredient at the ends of cell membrane glycoproteins and glycolipids, in addition to improving skin elasticity and moisturizing effect. Antioxidants and skin health are areas of interest to all ages, and the functionality of health functional food materials has been consistently prioritized. As the skin ages, the number of elastic fibers and the skin regeneration rate decrease and wrinkles increase. In addition, active oxygen generated as a by-product of metabolic activity, harmful external substances, and exposure to ultraviolet rays may damage the elastic fibers, advancing skin aging. Food intake can improve skin health; however, daily meals are often deficient in nutrients, and eating only certain foods can be detrimental to health. Epidermal growth factor (EGF) has been found in EBN, which has been proposed to correspond with the proliferative effect of EBN in epidermal tissues (Kong et al., 1987). In addition, N-acetylneuraminic acid, which is present in EBN, possesses a skin-whitening function (Chan et al., 2015), and additionally, EBN was shown to reduce water loss, wrinkle area, and dermal thickness of skin (Terazawa and Shimoda, 2020). Herein, we have provided a different evidence to reveal the signaling pathway of EBN extract and its regulation of the expressions of filaggrin and filaggrin-2, two important skin barrier proteins of the SFTP family playing roles in water balance of the skin surface. The EBN-mediated regulation of filaggrin and filaggrin-2 is demonstrated to be triggered by the p38-MAPK signaling pathway and various transcriptional factors, for example, GATA3, PPARα, PPARβ, and PPARγ (Lai et al., 2021).Therefore, we conducted this clinical trial to explore the effect of EBN on improving skin wrinkles, elasticity, moisture, and whitening to obtain evidence of the extract as a functional material for skin health.

## Methods and Design

### Study Participants

We recruited healthy participants through a written notice posted on the hospital’s homepage and bulletin board of the Chungbuk Cosmetics Clinical Research Support Center (Cheongju, Chungcheongbuk-do, the Republic of Korea) until the target sample size was reached. Women willing to participate in the study voluntarily visited the Chungbuk Cosmetics Clinical Research Support Center. After obtaining informed consent, we enrolled women who met the inclusion criteria. The first participant was enrolled in January 2021. The duration of the recruitment period was 8 months. The total sample size was 105. This clinical trial protocol (IRB No. SMCJH 2021-06) was approved by the Institutional Review Board of the Chungju Semyung University Korean Medicine Hospital and registered at the Korean Clinical Research Information Service (KCT0006558). Details of the eligibility and exclusion criteria are provided in Table 1.

**TABLE 1:** Eligibility and exclusion criteria.

Eligibility criteria
1) Women aged 40–60 years
2) Women with wrinkles around their eyes and a Global Photo Damage Score of 2–6
3) Women not currently consuming health functional foods or similar products for improving skin health, such as skin wrinkles, elasticity, moisturize, and whitening
4) Able to provide written informed consent
**Exclusion criteria**
1) Women with severe acute kidney disease, heart disease, liver disease, or other chronic diseases that may affect the test results within the last 6 months
2) Women with a history of stroke or transient ischaemic heart attack
3) Women with a history of malignancy or lung disease within 5 years of screening
4) Women with uncontrolled hypertension (systolic blood pressure >160 mmHg or diastolic blood pressure >100 mmHg, measured after 10 min of rest) or uncontrolled diabetes (fasting blood sugar >180 mg/dl)
5) Women with a neurological or psychological history or currently suffering from schizophrenia, epilepsy, alcoholism, drug addiction, anorexia, or bulimia
6) Women with skin abnormalities, such as spots, acne, tattoos, scars, erythema, capillary dilatation, and burn marks on the measurement site
7) Women who have consumed oral or applied retinoids/steroids within 6 months prior to the beginning of the test, or who have undergone skin peeling or skin wrinkle removal procedures
8) Women with irritation or allergy to cosmetics, pharmaceuticals, health functional foods, and daily exposure to sunlight
9) Women with a history of hypersensitivity (allergy) to bird nest extract
10) Pregnant women, lactating women, planning for pregnancy, and those under contraceptives or female hormones
11) Women receiving other prohibited treatment, such as insulin, antidepressant, antiserotonin, barbiturate, antipsychotic, drug with potential for abuse, absorption inhibitor, and appetite suppressant
12) Women who have consumed steroid-containing skin external agents for ≥2 weeks for the treatment of skin diseases
13) Women who have consumed steroids (oral and injection), hormones, or drugs that affect the absorption, distribution, metabolism, and the excretion of drugs within the past 3 months, and drugs that may affect the skin
14) Women with abnormalities in the general opinion of the specialist because of blood and urine tests, namely, AST, ALT, ALP, γ-GT, and total bilirubin levels greater than thrice UNL, and serum creatinine levels greater than twice the UNL
15) Within 3 months of participating in the clinical trial for skin health
16) Others considered unsuitable for the study at the discretion of the principal investigator

### Interventions

**FIGURE 1:**
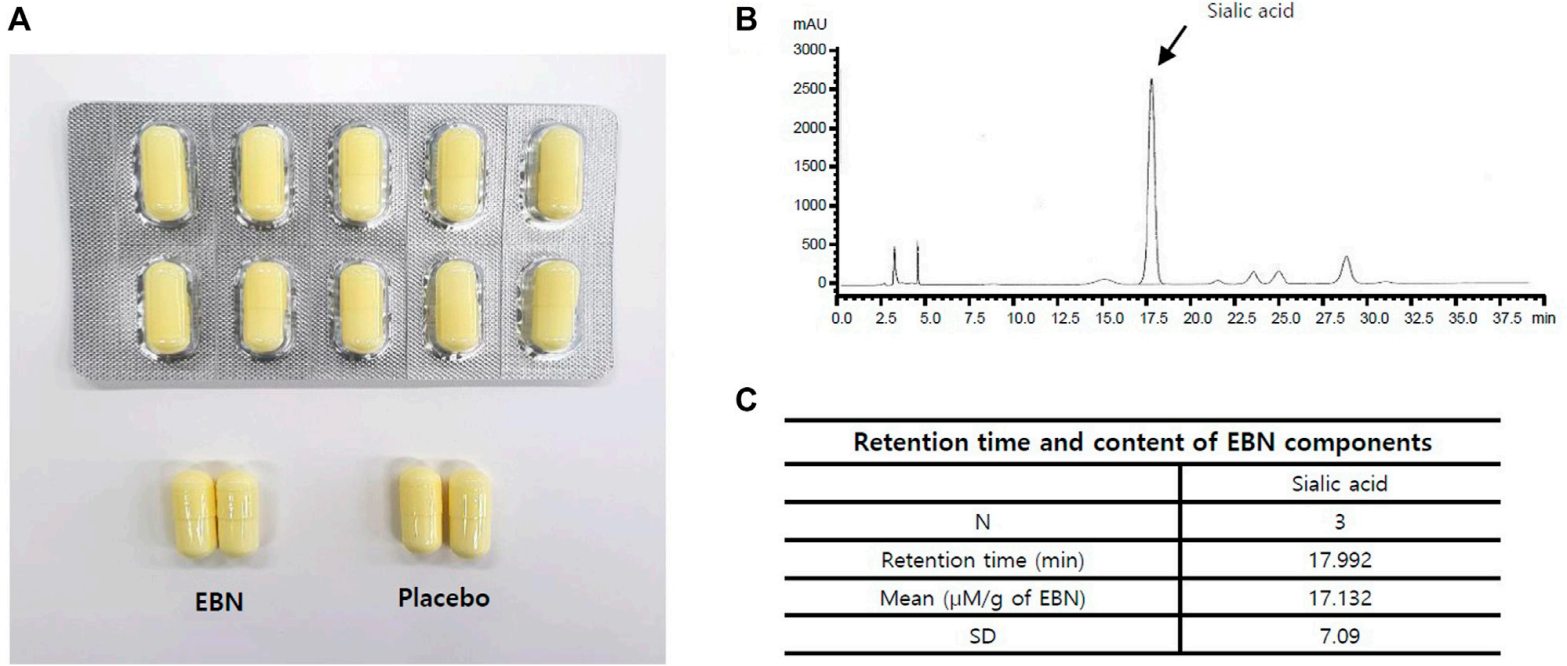
**(A)** EBN tablet and placebo. **(B,C)** Fingerprinting analysis of EBN. **(B)** Chromatogram of sialic acid HPLC specifications (x: time, y: sialic acid concentration). **(C)** The retention time of sialic acid in EBN components.

EBN extract, the primary ingredient of the test food, was obtained from Japan’s Oryza Oil & Fat Chemical Co., Ltd. and is similar to the product commercially sold by EBN extract-PK, which contains 20% of the hydrolyzed swiftlet nest extract. Therefore, one capsule of this test food contains 20 mg of the hydrolyzed swiftlet nest extract (Supplementary Table S2). The HPLC analysis for sialic acid was included in the test food and is presented in Supplementary Table S1 and Figures 1B,C. The active and placebo capsules were identical in shape, size, and color (Figure 1). The standardization of this test food capsule was guaranteed by strict process controls during manufacture and analysis of the hydrolyzed bird’s nest extract. EBN extract is a powder that is dried by adding maltodextrin after enzymatic treatment of edible bird’s nest with protease; the difference is that it has been subjected to enzyme treatment to increase absorption efficiency in the body and has been standardized with sialic acid, an indicator and functional ingredient (Kim et al., 2021). The participants were orally administered either an active or placebo tablet per dose once a day for 12 weeks. They received a 1-month supply of the investigational product at baseline and during weeks 4 and 8, following which they were encouraged to continue the prescribed dosage regimen. At weeks 4, 8, and 12, the unused capsules were returned and counted to evaluate participant compliance. We advised the participants to maintain their usual diet and exercise levels during the study. They were prohibited from consuming medicines or foods that could affect their skin during the study, and during the screening visit (visit 1), the subjects taking such medicines or foods were excluded so as not to affect the results of the outcome. Medicines, foods, exercise therapy, and diets maintained prior to their enrollment were allowed at the discretion of the principal investigator. Information regarding all concomitant medications, including the product or ingredient name, dosage, and duration, was recorded at every visit. We discontinued the intervention under the following conditions: a severe adverse event; a participant had used a drug or underwent a physical procedure that could affect the skin; a participant wished to discontinue study participation; difficulties in assessment for administrative reasons, such as violations of the dosage method or the visit schedule; and difficulties in follow-up owing to personal reasons.

### Randomization and Blinding

In this clinical trial, we performed block randomization. During screening, participants who met the eligibility criteria and were judged suitable for the clinical trial were assigned to the EBN and placebo groups. The randomization table is a sequence of random number permutations generated by the randomization program of the SAS 9.4 (SAS Institute, Cary, NY, the United States), beginning from number 1 for human subjects. The sponsor attached the food label for the human application test according to the randomization table during food packaging and supplied it to the testing institution before the commencement of this clinical trial.

The randomization codes were not disclosed until the end of the trial, except when it was unavoidably necessary to read the code. The clinical trial tester supplied the intervention that matched the registration number assigned to the selected participant.

### Outcome Measures and Endpoints

The primary endpoint comprised changes in skin wrinkles (in R1: skin roughness, R2: maximum roughness, R3: average roughness, R4: smoothness depth, and R5: arithmetic roughness average), skin elasticity (in R2: total elasticity, R5: real elasticity, and R7: the ratio of elasticity to the entire curve), and skin moisture, each after 12 weeks from the baseline.

The secondary endpoint comprised changes in skin wrinkles (in R1, R2, R3, R4, and R5), skin elasticity (in R2, R5, and R7), and skin moisture, each at 4 and 8 weeks from the baseline. Moreover, it comprised changes in the amount of transdermal water loss, melanin and erythema index, and visual evaluation of skin wrinkles measured by experts, each at 4, 8, and 12 weeks from the baseline. The visual evaluation was analyzed as one result by discussion among two experts.

Experts performed a visual evaluation of skin wrinkles at visits 1 (screening visit), 2 (week 0), 3 (week 4), 4 (week 8), and 5 (week 12), and after visits 1 and 2. The results of visit 1 could be recorded for visit 2 conducted within a week. At visit 1, the selection criteria were confirmed by identifying the presence of wrinkles around the eyes and the Global Photo Damage Score (approximately 2–6 points), visually or by photographs. The SV700 evaluated skin wrinkles, the Cutometer MPA 5809 evaluated skin elasticity values, the Corneometer CM 825 evaluated skin moisture values, the Tewameter TM 300 evaluated transdermal moisture loss, and the Mexameter MX 18 evaluated skin whitening. The evaluation time comprised four visits, including baseline, visit 3 (week 4), visit 4 (week 8), and visit 5 (week 12). Skin wrinkle evaluation using the SV700 measured the area 2–3 cm away from the left eye, and the remaining techniques measured the right-angle intersection of the corner of the eye and the tip of the nose. The test site was kept clean and dry to ensure similar measurement conditions in all participants. In addition, the skin was stabilized in a place with constant temperature and humidity (22 ± 2°C, R.H. 40−60%) for at least 30 min before proceeding. Water intake was restricted 1 h before the measurement.

### Safety

Safety was evaluated through adverse reactions, laboratory tests (hematology, blood chemistry, and urine), and vital signs (systolic blood pressure, diastolic blood pressure, pulse, and body temperature).

### Sample Size

We referred to the article by Hwang et al. (2015) and used the amount of change in the average depth of skin wrinkles (R4 smoothness depth) using a micro-wrinkle analyzer. More than 104 people who met the inclusion and exclusion criteria were recruited to be allocated to the investigational product for human clinical research. Furthermore, it was planned to analyze more than 41 people per group with the number of subjects who completed the study as per the protocol and have no major protocol deviations that may affect the interpretation of the primary endpoint.

The formula for calculating the number of subjects in the human application test was as follows:

$$n_{c}=\frac{2{\left(Z_{1-\alpha /2}+Z_{1-\beta }\right)}^{2}\sigma^{2}}{{\left(D_{t}-D_{c}\right)}^{2}}\;=\;\frac{2{\left(1.960+1.036\right)}^{2}0.03^{2}}{{\left(-0.02\right)}^{2}}\;\approx 41,$$

H0 :Dt−Dc=0   vs  H1 :Dt−Dc≠0.

$D_{t}$ : Amount of change in R4 after the ingestion of BNE

$D_{c}$ : Amount of change in R4 after the ingestion of placebo

### Statistical Analyses

A statistical hypothesis test was conducted at a significance level of 0.05 (two-sided). The number of subjects, mean, and standard deviation were presented for primary and secondary endpoints. In addition, depending on whether the data normality assumption was satisfied, analysis of covariate (ANCOVA) used baselines as a covariate, or the Wilcoxon rank-sum test was performed to compare the difference between the groups. For demographic characteristics and laboratory test results, mean and standard deviation were presented, and a two-sample t-test or the Wilcoxon rank-sum test was used for inter-group comparison for continuous data. However, for categorical data, frequency and percentage were calculated, and Pearson’s chi-square test or Fisher’s exact test was used for inter-group comparison. Intra-group analysis was performed using the paired t-test or the Wilcoxon signed-rank test. Per-protocol set was used as the analysis for primary and secondary endpoints. Per-protocol set included all subjects who completed the study per the protocol and had no major protocol deviations. Moreover, a safety set that included all subjects who received at least one capsule of the investigational product and had at least one safety assessment was used as the safety analysis.

## Results

For this clinical trial, the first participant for the human application trial was screened on 11th January 2021, whereas the last participant was screened on 23rd April 2021.

**FIGURE 2:**
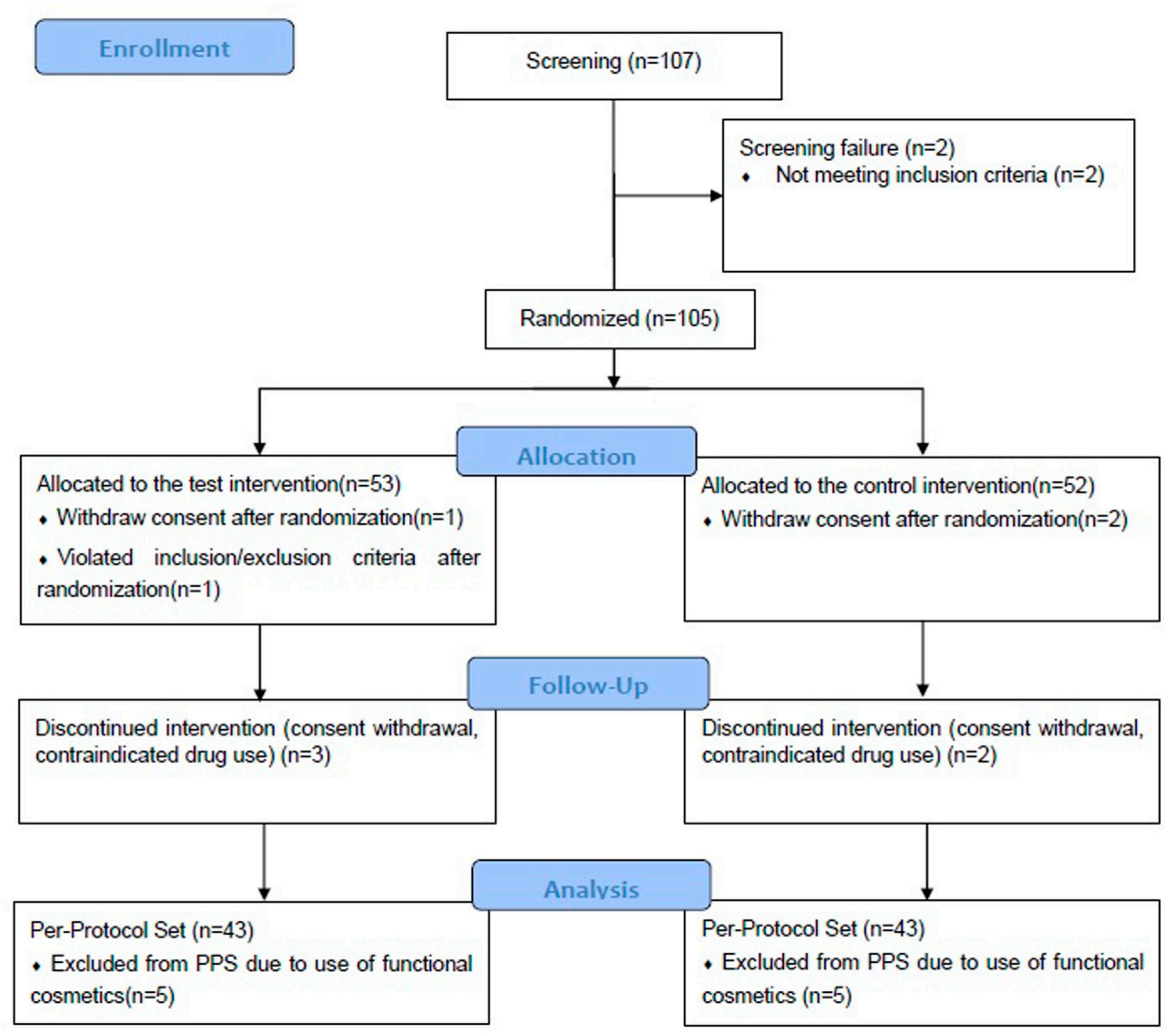
Study flowchart. Evaluation*: Adverse reactions and vital signs check, laboratory test (safety evaluation), pregnancy test, drug history/concomitant drug examination, efficacy evaluation, and skin functional test.

A total of 105 participants were enrolled for the clinical trial, and the per-protocol set comprised 86 participants (Table 2), 43 each in the EBN and placebo groups (Figure 2 and Supplementary Table S3).

**TABLE 2:** Demographic characteristics of subjects.

		EBN (n = 43)	Placebo (n = 43)	*p*-Value
Mean age (year)		46.1	46.8	0.3762*
Mean height (cm)		161.1	160.1	0.3380*
Mean weight (kg)		59.5	60.7	0.4969*
Dietary habit (regular/irregular)		37/6	35/8	0.5591^†^
Exercise (times/week)	None	19	18	0.6135^‡^
	1–2	14	12	
	3–4	7	12	
	5–6	2	1	
	Everyday	1	0	
Smokers (non-smoker/ex-smoker/smoker)	42/0/1		41/1/1	1.0000^‡^
Drinking (non-drinker/quit drinking/current drinker)	31/1/11		34/0/9	0.6164^‡^

*Two-sample t-test.

^†^Chi-square test.

^‡^Fisher’s exact test.

### Primary Outcome

**FIGURE 3:**
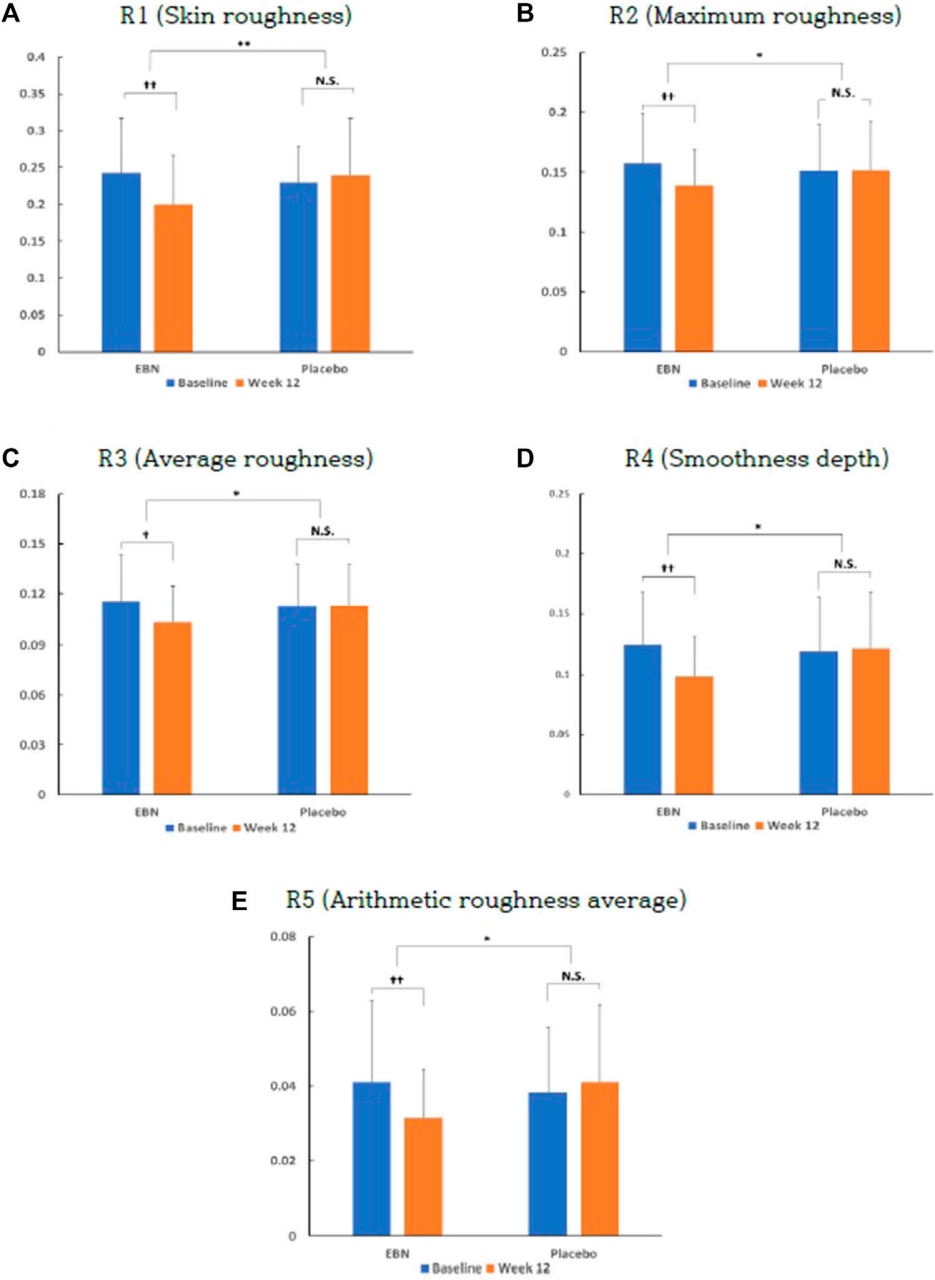
Effect of EBN on skin wrinkle parameters at baseline and week 12. Data are presented as the means ± SD. *Indicates that the *p*-value between the two groups is <0.05. **Indicates that the *p*-value between the two groups is <0.01. ^†^Indicates that the *p*-value compared within groups is <0.05. ^†^ indicates that the *p*-value compared within groups is <0.01. N.S. is an abbreviation for not significant.

The EBN group showed a significant decrease in the skin wrinkle value compared to the placebo group at baseline and week 12. Elasticity decreased in both the groups; however, there was no statistical significance. Regarding the amount of skin moisture change before and after intake, there was no statistically significant difference between the EBN and the placebo groups (Figure 3 and Supplementary Table S4).

### Secondary Outcomes

The skin elasticity value (R2: total elasticity, R5: real elasticity, and R7: ratio of elasticity to the entire curve) had decreased in both the EBN and placebo groups at baseline and week 12 (Supplementary Table S4).

Compared to the previous reading, there was no tendency for decrease or increase in the skin wrinkle value, skin elasticity value, and skin moisture value at week 4 and week 8 (Supplementary Table S5).

The change in transdermal water loss displayed a tendency to increase, and the melanin index and erythema index decreased in both groups at baseline and week 12 (Supplementary Table S6). However, there was no significant difference in secondary outcomes between the two groups.

### Adverse Reaction

No adverse reactions occurred during this study. There was no minimally detectable change or minimal clinically significant change among the vital sign variables. Laboratory test results (hematology, blood chemistry, and urine) showed minimal clinically significant changes (Supplementary Table S7).

## Discussion

EBN is a swiftlets’ (*Aerodramus fuciphagus*) residence made of their salivary gland secretions mixed with feathers or grass during breeding (Kew et al., 2014; Dai et al., 2021). The largest number of EBN was often found in caves or houses in Southeast Asia and the South Pacific, depending on the habitat distribution (Kew et al., 2014; Daud et al., 2021). EBN has been regarded as a high-grade healthy food that only privileged individuals can eat, with functions of moistening the lung, nourishing the stomach, relieving the liver, clearing the eyes, and tonifying the heart (Hwang et al., 2015). It is composed of protein, carbohydrate, ash, fat, and dietary elements (Quek et al., 2018), and has various effects, including antiviral effects and inhibitory hemagglutination (Guo et al., 2017); immunological enhancement (Zhao et al., 2016); improving intelligence and memory (Oliveros et al., 2018); improvement of neurodegenerative disease (Yew et al., 2014; Careena et al., 2018) and cardiovascular disease (Hun Lee et al., 2020); promoting cell division (Roh et al., 2012); antioxidant, anti-inflammatory, and anti-aging effects (Yida et al., 2015; Hun Lee et al., 2020) on epidermal growth factor-like activity (Kong et al., 1987); and stimulating collagen production and epithelial tissue proliferation (Park et al., 2017). EBN is expected to have various effects on skin health through these effects. Therefore, we tried to investigate the effect of EBN extract on skin wrinkles and whitening. We conducted this randomized, double-blind study to determine the effect of EBN extract on improving skin wrinkles, elasticity, moisture, and whitening. In a previous experimental study, the authors confirmed the safety of the extract (Haghani et al., 2016).

We observed a causal relationship between EBN and skin wrinkle improvement. We compared the amount of change in skin wrinkle (R1, R2, R3, R4, and R5) before (0 weeks) and after (12 weeks) the intake of EBN extract with the R1 value measured before the intake. Afterward, it was confirmed that the EBN group had significantly decreased the skin wrinkle value compared to the placebo group at baseline and week 12. In addition, R2, R3, R4, and R5 decreased significantly in the EBN group compared with those in the placebo group. In addition, the EBN and placebo groups demonstrated a statistically significant decrease in the skin wrinkle value before and after 12 weeks of the intervention. As a result, it was demonstrated that consumption of EBN extract positively affects skin wrinkle improvement.

There was no significant difference in secondary outcomes between the two groups. The skin elasticity value (R2: total elasticity, R5: real elasticity, and R7: ratio of elasticity to the entire curve) was decreased in both the EBN and placebo groups at baseline and week 12. The amount of change in transdermal water loss displayed a tendency to increase in both groups at baseline and week 12, and the melanin index and erythema index decreased in both groups at baseline and week 12.

There are studies that have documented that EBN containing EGF promotes and activates the synthesis of DNA, and thus promotes cell proliferation (Kong. et al., 1987). A further experiment was conducted to study the effects of EBN extract, with rich EGF, on human skin cell (epidermal keratinocytes and dermal fibroblasts) proliferation. The results showed that EBN extract promoted the proliferation of normal keratinocytes and fibroblasts dose-dependently (Kong et al., 1987). Keratinocytes play an important role in healthy skin barrier function, improved proliferation of keratinocytes directly improves skin barrier function, thus promoting skin suppleness and improving the overall skin texture. Meanwhile, fibroblasts synthesize extracellular matrix and collagen, and play a critical role in wound healing, influence skin elasticity, and physically apparent aging.

EGF plays an important role in the process of wound healing. Studies showed that wound healing is delayed in mice whose submandibular gland is removed as the submandibular gland produces EGF (Niall et al., 1982). Proliferation and migration of epithelial cells play a critical role in wound healing, and EGF is a known factor in promoting fibroblast proliferation and epithelial cells migration, thus promoting wound healing. The effect of EBN extract on wound healing was examined by a scratch test. Normal human keratinocytes were cultured in a petri dish. A damage model was induced by scratching a line in the center of the dish. Wound healing at the scratched area was observed after 24-h. Accelerated wound healing effect was observed in the dish treated with EBN extract, and the effect was dose-dependent. Thus, it is suggestive that EBN extract promotes wound healing (Niall et al., 1982).

Tight junctions (TJs) refer to a closely associated area of two adjacent cells whose membrane join together forming a virtually impermeable barrier to fluid. In the epidermis, TJs play a crucial role in the formation and maintenance of epithelial barriers and prevent invasion of foreign particles. In the stratum corneum, ceramide acts as a first line of skin barrier. TJs in the stratum granulosum act as the second line of skin barrier. Occludin and claudin are integral plasma membrane proteins located at the TJs (Niessen, 2007). They form important barrier that protect the skin from external organism, prevent excessive water loss, and selectively transport small solutes through the skin (Yamamoto et al., 2008). This has prompted further understanding into the effect of EBN on skin barrier function where an experiment was conducted to evaluate the effect of EBN on skin TJs. Among the TJ proteins, claudin-1 (Cldn-1) and claudin-4 (Cldn-4) have been demonstrated to have a role in skin barrier function (Furuse et al., 2002; Yuki et al., 2011). The effect of EBN on the expression of TJ proteins was examined. Both genetic expression and protein expression of Cldn-1 and Cldn-4 was upregulated by EBN-P (PC) 0.1% *in vitro*. In addition, the effect of EBN on the protein expression of Cldn-4 of normal keratinocytes was examined and observed using fluorescence microscopy. The results showed that the protein expression of Cldn-4 was upregulated by EBN-P (PC) 0.1%.

Sialic acid is the major carbohydrate found in EBN and in several tissues and fluids in humans (Kim et al., 2021). A study demonstrated the antioxidant role of sialic acid as a hydroxyl radical scavenger (Ogasawara et al., 2007), and another study found that sialic acid from EBN has the effects of scavenging DPPH radicals and hydroxyl radicals (Wang et al., 2019). Several studies have demonstrated that sialic acid is essential for brain function, immune function, and cell proliferation and repair (Wang, 2009; Varki and Gagneux, 2012; Rashed et al., 2021); however, there was no statistically significant difference between the two groups in the amount of skin moisture change. Considering that the present trial was conducted during the COVID-19 pandemic, all participants routinely used medical masks. Therefore, the increase in skin moisture content and transdermal moisture loss was a possible outcome of using masks for a prolonged period. Furthermore, this is a single-center study, not a cross-over study; therefore, it is highly likely that only subjects from a specific region had participated, which may lead to consequential bias. It can be seen that there was a slightly negative effect as the mask dries the skin, which can lead to wrinkles and loss of moisture in the skin. In contrast, we evaluated skin wrinkles at a position 2–3 cm away from the left eye, which was not affected by long-term mask-wearing; therefore, it is considered an objective functional evaluation index and can be evaluated by excluding other external factors (wearing a mask). There were no adverse reactions or clinically meaningful changes in the laboratory test values following the ingestion of EBN extract. The consumption of EBN extract for 12 weeks objectively confirmed skin wrinkle improvement and a tendency to increase skin moisture and decrease the melanin and erythema index. In addition, there were no safety-associated problems. Therefore, EBN extract was effective in improving skin wrinkles. Moreover, it is beneficial for skin health and can be used as a skin nutritional supplement.

## Conclusion

In this study, upon comparing changes in skin wrinkle values between the two intake groups at 12 weeks of ingestion, those in the EBN extract group decreased significantly compared with the placebo group. However, the skin elasticity value decreased and skin moisture value increased, but there was no statistical significance between the two groups. The change in transdermal water loss displayed a tendency to increase, and the melanin index and erythema index decreased in both groups at baseline and week 12. However, there was no significant difference in the secondary outcomes between the two groups.

No adverse reactions were recorded during the study. In the case of laboratory test results, changes before and after ingestion of the test food were within the normal range, and there was no minimally detectable change or minimal clinically significant change.

This study has several limitations, including that this is a single-center study, not a cross-over study. Moreover, there is no verum group in the study. However, if multicenter and cross-over studies are conducted in the future, it is expected that clearer and more reliable research results will be obtained. A verum group should be included in the next study, where a follow-up phase should be performed to assess skin wrinkle values of the EBN group after a period of no drug treatment. Furthermore, in future studies, it is thought that research and statistical analysis on the skin improvement effect by age should be performed.

## Data Availability

The original contributions presented in the study are included in the article/**Supplementary Material**, further inquiries can be directed to SE: daehanchemtech@dhchemtech.com / SY: sjyoun@rnbs.co.kr / YC: choiy@rnbs.co.kr.
