# Supplementary material for: Anti-Wrinkle Efficacy of Edible Bird’s Nest Extract: A Randomized, Double-Blind, Placebo-Controlled, Comparative Study
Source: Front Pharmacol. 2022 Mar 9;13:843469. doi: 10.3389/fphar.2022.843469 (PMC8959461; doi:10.3389/fphar.2022.843469)
Supplement: Supplementary file 1 [file DataSheet1.docx]

Supplementary Material

**Supplementary Table 1. HPLC method (**Analytical instrument: HPLC-FLD)

| 1. Preparation of the analytical sample solution  (1) Accurately measure about 10 mg of the sample and add 0.025 M of 0.5 µL sulfuric acid to complete dissolution. |
| --- |
| 2. Release of sialic acid by acid hydrolysis  (1) Heat the sample prepared in step 1 in 80 °C water for 1 hour.  (2) Put the complete sample in a 50 mL volumetric flask and use distilled water. |
| 3. Preparation of standard solution of sialic acid (N~acetylneuraminic acid=Neu5Ac)  (1) Measure 60 µL of distilled water in a 5 mL microtube with a pipette, add 20 µL of Neu5Ac standard (100 µM) attached to the reagent kit for sialic acid fluorescence labelling with a pipette, and mix well (25 µM). Dilute it with distilled water to prepare a standard solution. |
| 4. Fluorescent labelling of sialic acid by 1,2-Diamino~4,5-methylenedioxybenzene (DMB)  (1) Prepare a mixture of DMB solution (reagent 1) and coupling solution (reagent 2) attached to the sialic acid fluorescent labelling kit in the ratio of reagent 1: reagent 2: distilled water = 1: 5: 4.  (2) Measure 20 µL of the sample solution in step 2 and the standard solution in step 3 with a pipette and place it in a 2.0 screw cap tube.  (3) Measure and add 200 µL of the mixture solution from step (1) to the screw cap tube from step (2) with a pipette, and mix with a vortex.  (4) Put the screw cap tube from step (3) in a water bath at 50 °C and block the light and react for 2 hours and 30 minutes.  (5) Cool the screw cap tube from step (4) (5 minutes), complete the reaction, and then stir with a vortex.  (6) Filter the solution from the screw cap tube from step (5) with a syringe filter (0.45 mm) and put it in an HPLC vial. |

HPLC is an abbreviation for High Performance Liquid Chromatography

**Supplementary Table 2**. Intervention composition

| **EBN** | | | **Placebo** | | |
| --- | --- | --- | --- | --- | --- |
| **Raw material** | **Mixing ratio (%)** | **Content (mg)** | **Raw material** | **Mixing ratio (%)** | **Content (mg)** |
| EBN^1^ extract | 20.222 | 100.00 | Crystalline cellulose | 20.222 | 100.00 |
| Maltodextrin | 74.778 | 336.50 | Maltodextrin | 74.778 | 336.50 |
| Magnesium Stearate | 1.500 | 6.75 | Magnesium Stearate | 1.500 | 6.75 |
| Silicon dioxide | 1.500 | 6.75 | Silicon dioxide | 1.500 | 6.75 |
| Sum | 100.00 | 450.00 | Sum | 100.00 | 450.00 |

EBN is an abbreviation for Edible Bird's Nest

**Supplementary Table 3**. Test schedule

| Test items | | | Screening | Intake period | | | | F/U  (If necessary) |
| --- | --- | --- | --- | --- | --- | --- | --- | --- |
|  |  |  | Visit 1 | Visit 2 | Visit 3 | Visit 4 | Visit 5 |  |
|  |  |  | -2 weeks | 0 week | 4 weeks | 8 weeks | 12 weeks | 14 weeks |
|  |  |  | (-14–1 days) | (0 day) | (28±5 days) | (56±5 days) | (84±5 days) | (98±5 days) |
| Subject consent | | | O |  |  |  |  |  |
| Demographic survey | | | O |  |  |  |  |  |
| Vital signs | | | O | O | O | O | O | O |
| Medical history and disease investigation | | | O |  |  |  |  |  |
| Prior/Concomitant Investigation | | | O | O | O | O | O | O |
| Laboratory examination^1^ | | | O | O |  |  | O | O |
| Pregnancy test (HCG) | | | O | O | O | O | O | O |
| Cosmetics and skin care device investigation | | | O | O | O | O | O | O |
| Subject suitability evaluation | | | O |  |  |  |  |  |
| Randomisation | | |  | O |  |  |  |  |
| Skin Functional test | Skin wrinkles | Expert Visual Assessment |  | O | O | O | O | O |
|  |  | SV700 |  | O | O | O | O | O |
|  | Skin elasticity cutometer | |  | O | O | O | O | O |
|  | Skin moisture corneometer | |  | O | O | O | O | O |
|  | Transdermal water loss tewameter | |  | O | O | O | O | O |
|  | Skin whitening mexameter | | O | O |  |  | O | O |
| Intervention provision | | |  | O | O | O |  |  |
| Investigation of adverse reactions | | |  |  | O | O | O | O |
| Returned intervention and evaluation of compliance | | |  |  | O | O | O | O |
| 1.Laboratory examination:   1. Complete blood count: White blood cells, red blood cells, haemoglobin, haematocrit, platelets, neutrophils, lymphocytes, monocyte, eosinophils, and basophils 2. Biochemistry: aspartate aminotransferase, alanine transaminase, gamma-glutamyl transferase, alkaline phosphatase, bilirubin-total, total protein, albumin, triglycerides, high-density lipoprotein cholesterol, low-density lipoprotein cholesterol, blood urea nitrogen, creatinine, uric acid, glucose, C-reactive protein, and erythrocyte sedimentation rate 3. Urine test: pH, protein, glucose, WBC, RBC, and specific gravity | | | | | | | | |

**Supplementary Table 4**. Skin wrinkle, elasticity, and moisture value at baseline and week 12

|  |  | EBN (n=43) | | | |  | Placebo (n=43) | | | | *p*-value^1^ |
| --- | --- | --- | --- | --- | --- | --- | --- | --- | --- | --- | --- |
|  |  | Baseline | | After 12 wk | |  | Baseline | | After 12 wk | |  |
| Skin wrinkle value (A.U.) | | |  |  |  |  |  |  |  |  |  |
|  | R1 | 0.2426 | ±0.07 | 0.2002 | ±0.05 |  | 0.2295 | ±0.07 | 0.2395 | ±0.08 | 0.0012^‡^ |
|  | R2 | 0.1574 | ±0.04 | 0.1391 | ±0.03 |  | 0.1514 | ±0.04 | 0.1519 | ±0.04 | 0.0338^†^ |
|  | R3 | 0.1153 | ±0.03 | 0.104 | ±0.02 |  | 0.1126 | ±0.03 | 0.1128 | ±0.02 | 0.0286^†^ |
|  | R4 | 0.1249 | ±0.04 | 0.0988 | ±0.03 |  | 0.1195 | ±0.04 | 0.1221 | ±0.05 | 0.0126^‡^ |
|  | R5 | 0.0412 | ±0.02 | 0.0316 | ±0.01 |  | 0.0384 | ±0.02 | 0.0412 | ±0.02 | 0.0238^‡^ |
|  |  |  |  |  |  |  |  |  |  |  |  |
| Skin elasticity value (A.U.) | | |  |  |  |  |  |  |  |  |  |
|  | R2 | 0.7031 | ±0.07 | 0.6581 | ±0.06 |  | 0.6908 | ±0.07 | 0.663 | ±0.05 | 0.4263^†^ |
|  | R5 | 0.6527 | ±0.08 | 0.6258 | ±0.06 |  | 0.6527 | ±0.09 | 0.6338 | ±0.06 | 0.4762^†^ |
|  | R7 | 0.5159 | ±0.06 | 0.4878 | ±0.05 |  | 0.5084 | ±0.07 | 0.4924 | ±0.05 | 0.4248^†^ |
|  |  |  |  |  |  |  |  |  |  |  |  |
| Skin moisture value (A.U.) | | |  |  |  |  |  |  |  |  |  |
|  |  | 64.72 | ±7.16 | 66.37 | ±7.90 |  | 67.53 | ±7.66 | 69.73 | ±6.75 | 0.6880^‡^ |

^1^Comparison between groups, ^†^ANCOVA, ^‡^Wilcoxon rank sum test

‘A.U.’ is an abbreviation for arbitrary unit. In the skin wrinkle value, R1 corresponds to skin roughness, R2 corresponds to maximum roughness, R3 corresponds to average roughness, R4 corresponds to smoothness depth and R5 corresponds to arithmetic roughness average. In the skin elasticity value, R2 corresponds to overall elasticity of the skin including creep and creep recovery (Ua/Uf), R5 corresponds to net elasticity (Ur/Ue) and R7 corresponds to ratio of elastic recovery to the total deformation (Ur/Uf). Ua (Total retraction, mm), Ue (Immediate distension, mm), Uf (Total elongation, mm), Ur (Immediate retraction, mm). EBN is an abbreviation for Edible Bird's Nest

**Supplementary Table 5**. Skin wrinkle, elasticity, and moisture value at baseline, week 4, and week 8

|  |  | Baseline | |  | After 4 wk | |  | After 8 wk | | *p*-value^1^ | |
| --- | --- | --- | --- | --- | --- | --- | --- | --- | --- | --- | --- |
|  |  | mean | ±SD |  | mean | ±SD |  | mean | ±SD |  |  |
| Skin wrinkle value (A.U.) | |  |  |  |  |  |  |  |  |  |  |
| R1 | EBN (n=43) | 0.2426 | ±0.07 |  | 0.2370 | ±0.07 |  | 0.2447 | ±0.07 | 4 wk: | 0.9202^†^ |
|  | Placebo (n=43) | 0.2295 | ±0.07 |  | 0.2295 | ±0.07 |  | 0.2381 | ±0.08 | 8 wk: | 0.4783^‡^ |
|  |  |  |  |  |  |  |  |  |  |  |  |
| R2 | EBN (n=43) | 0.1574 | ±0.04 |  | 0.1560 | ±0.04 |  | 0.1556 | ±0.04 | 4 wk: | 0.9036^†^ |
|  | Placebo (n=43) | 0.1514 | ±0.04 |  | 0.1514 | ±0.05 |  | 0.1519 | ±0.04 | 8 wk: | 0.7908^†^ |
|  |  |  |  |  |  |  |  |  |  |  |  |
| R3 | EBN (n=43) | 0.1153 | ±0.03 |  | 0.1147 | ±0.03 |  | 0.1137 | ±0.02 | 4 wk: | 0.7113^‡^ |
|  | Placebo (n=43) | 0.1126 | ±0.03 |  | 0.1100 | ±0.03 |  | 0.1119 | ±0.03 | 8 wk: | 0.8069^†^ |
|  |  |  |  |  |  |  |  |  |  |  |  |
| R4 | EBN (n=43) | 0.1249 | ±0.04 |  | 0.1228 | ±0.04 |  | 0.1244 | ±0.05 | 4 wk: | 0.9200^†^ |
|  | Placebo (n=43) | 0.1195 | ±0.04 |  | 0.1193 | ±0.04 |  | 0.1179 | ±0.05 | 8 wk: | 0.9345^‡^ |
|  |  |  |  |  |  |  |  |  |  |  |  |
| R5 | EBN (n=43) | 0.0412 | ±0.02 |  | 0.0393 | ±0.02 |  | 0.0407 | ±0.02 | 4 wk: | 0.2477^‡^ |
|  | Placebo (n=43) | 0.0384 | ±0.02 |  | 0.0395 | ±0.01 |  | 0.0414 | ±0.02 | 8 wk: | 0.6461^‡^ |
|  |  |  |  |  |  |  |  |  |  |  |  |
| Skin elasticity value (A.U.) | |  |  |  |  |  |  |  |  |  |  |
| R2 | EBN (n=43) | 0.7031 | ±0.07 |  | 0.6612 | ±0.06 |  | 0.6717 | ±0.05 | 4 wk: | 0.8329^†^ |
|  | Placebo (n=43) | 0.6908 | ±0.07 |  | 0.6521 | ±0.07 |  | 0.6663 | ±0.05 | 8 wk: | 0.2842^‡^ |
|  |  |  |  |  |  |  |  |  |  |  |  |
| R5 | EBN (n=43) | 0.6527 | ±0.08 |  | 0.6137 | ±0.06 |  | 0.6195 | ±0.06 | 4 wk: | 0.8162^†^ |
|  | Placebo (n=43) | 0.6527 | ±0.09 |  | 0.6165 | ±0.08 |  | 0.6285 | ±0.06 | 8 wk: | 0.4083^†^ |
|  |  |  |  |  |  |  |  |  |  |  |  |
| R7 | EBN (n=43) | 0.5159 | ±0.06 |  | 0.4827 | ±0.06 |  | 0.4918 | ±0.05 | 4 wk: | 0.8511^†^ |
|  | Placebo (n=43) | 0.5084 | ±0.07 |  | 0.4766 | ±0.06 |  | 0.4898 | ±0.05 | 8 wk: | 0.9089^†^ |
|  |  |  |  |  |  |  |  |  |  |  |  |
| Skin moisture value (A.U.) | |  |  |  |  |  |  |  |  |  |  |
|  | EBN (n=43) | 64.72 | ±7.16 |  | 66.54 | ±7.39 |  | 66.21 | ±7.09 | 4 wk: | 0.3061^‡^ |
|  | Placebo (n=43) | 67.53 | ±7.66 |  | 68.65 | ±7.76 |  | 68.83 | ±7.03 | 8 wk: | 0.6816^‡^ |

^1^Comparison between groups, ^†^ANCOVA, ^‡^Wilcoxon rank sum test

Note: ‘4 wk’ means group comparison of difference between week 4 and baseline. ‘8 wk’ means group comparison of difference between week 8 and baseline. ‘A.U.’ is an abbreviation for arbitrary unit. In the skin wrinkle value, R1 corresponds to skin roughness, R2 corresponds to maximum roughness, R3 corresponds to average roughness, R4 corresponds to smoothness depth and R5 corresponds to arithmetic roughness average. In the skin elasticity value, R2 corresponds to overall elasticity of the skin including creep and creep recovery (Ua/Uf), R5 corresponds to net elasticity (Ur/Ue), and R7 corresponds to ratio of elastic recovery to the total deformation (Ur/Uf). Ua (Total retraction, mm), Ue (Immediate distension, mm), Uf (Total elongation, mm), and Ur (Immediate retraction, mm). EBN is an abbreviation for Edible Bird's Nest

**Supplementary Table 6**. Transdermal water loss amount, melanin index, erythema index, and GPDS at all-time points

|  |  | Baseline | |  | After 4 wk | |  | After 8 wk | |  | After 12 wk | | *p*-value^1^ | |
| --- | --- | --- | --- | --- | --- | --- | --- | --- | --- | --- | --- | --- | --- | --- |
|  |  | mean | ±SD |  | mean | ±SD |  | mean | ±SD |  | mean | ±SD |  |  |
| Transdermal water loss amount (g/m^2^/h) | | | |  |  |  |  |  |  |  |  |  |  |  |
|  | EBN (n=43) | 8.98 | ±2.41 |  | 8.90 | ±2.22 |  | 9.29 | ±2.62 |  | 9.43 | ±3.35 | 4 wk: | 0.8538^†^ |
|  | Placebo (n=43) | 8.14 | ±2.62 |  | 8.28 | ±2.94 |  | 8.04 | ±3.00 |  | 8.55 | ±3.08 | 8 wk: | 0.1420^‡^ |
|  |  |  |  |  |  |  |  |  |  |  |  |  | 12 wk: | 0.7330^‡^ |
| Melanin index (A.U.) | | |  |  |  |  |  |  |  |  |  |  |  |  |
|  | EBN (n=43) | 157.76 | ±27.83 |  | 156.78 | ±27.37 |  | 157.30 | ±26.98 |  | 157.05 | ±26.70 | 4 wk: | 0.2800^‡^ |
|  | Placebo(n=43) | 147.96 | ±25.11 |  | 145.87 | ±23.61 |  | 144.52 | ±22.43 |  | 145.18 | ±22.99 | 8 wk: | 0.0670^‡^ |
|  |  |  |  |  |  |  |  |  |  |  |  |  | 12 wk: | 0.0972^‡^ |
| Erythema Index (A.U.) | | |  |  |  |  |  |  |  |  |  |  |  |  |
|  | EBN (n=43) | 243.36 | ±58.20 |  | 233.00 | ±51.56 |  | 229.60 | ±53.15 |  | 236.84 | ±50.47 | 4 wk: | 0.4842^‡^ |
|  | Placebo (n=43) | 240.77 | ±53.24 |  | 234.57 | ±49.46 |  | 232.34 | ±54.24 |  | 244.20 | ±53.81 | 8 wk: | 0.4902^†^ |
|  |  |  |  |  |  |  |  |  |  |  |  |  | 12 wk: | 0.4395^‡^ |
| GPDS (grade) | |  |  |  |  |  |  |  |  |  |  |  |  |  |
|  | EBN (n=43) | 3.84 | ±1.04 |  | 3.86 | ±1.06 |  | 3.88 | ±1.05 |  | 3.77 | ±1.02 | 4 wk: | 1.0000^†^ |
|  | Placebo (n=43) | 3.53 | ±0.96 |  | 3.56 | ±0.96 |  | 3.65 | ±0.87 |  | 3.51 | ±0.86 | 8 wk: | 0.4775^†^ |
|  |  |  |  |  |  |  |  |  |  |  |  |  | 12 wk: | 0.7064^†^ |

^1^Comparison between groups, ^†^ANCOVA, ^‡^Wilcoxon rank sum test

‘4 wk’ means group comparison of difference between week 4 and baseline. ‘8 wk’ means group comparison of difference between week 8 and baseline. ‘12 wk’ means group comparison of difference between week 12 and baseline. ‘A.U.’ and ‘GPDS’ are abbreviations for arbitrary unit and global photo damage score, respectively. EBN is an abbreviation for Edible Bird's Nest

**Supplementary Table 7**. Laboratory test result

|  | EBN | | | |  | Placebo | | | | *p*-value^1^ |
| --- | --- | --- | --- | --- | --- | --- | --- | --- | --- | --- |
|  | Baseline (n=53) | | After 12 wk  (n=48) | |  | Baseline (n=52) | | After 12 wk  (n=48) | |  |
| Haematology, mean±SD |  |  |  |  |  |  |  |  |  |  |
| WBC (10³/µL) | 5.88 | ±1.30 | 6.09 | ±1.38 |  | 5.89 | ±1.71 | 5.94 | ±1.48 | 0.7031^‡^ |
| RBC (10^6^/µL) | 4.26 | ±0.31 | 4.20 | ±0.32 |  | 4.34 | ±0.31 | 4.27 | ±0.28 | 0.5805^†^ |
| Haemoglobin (g/dL) | 12.82 | ±1.47 | 12.67 | ±1.39 |  | 12.99 | ±1.27 | 12.81 | ±1.12 | 0.8903^†^ |
| Haematocrit (%) | 39.74 | ±3.71 | 39.44 | ±3.48 |  | 40.29 | ±3.15 | 39.76 | ±2.78 | 0.6034^†^ |
| Platelets (10³/µL) | 251.98 | ±64.14 | 251.19 | ±56.96 |  | 246.96 | ±52.28 | 243.94 | ±41.00 | 0.8089^‡^ |
|  |  |  |  |  |  |  |  |  |  |  |
| Blood chemistry, mean±SD |  |  |  |  |  |  |  |  |  |  |
| Glucose (mg/dL) | 84.57 | ±24.34 | 82.63 | ±6.98 |  | 84.50 | ±9.16 | 87.58 | ±7.73 | 0.5164^†^ |
| BUN (mg/dL) | 12.46 | ±3.34 | 12.73 | ±3.34 |  | 12.21 | ±2.76 | 12.96 | ±3.37 | 0.9825^‡^ |
| Creatinine (mg/dL) | 0.66 | ±0.07 | 0.67 | ±0.08 |  | 0.65 | ±0.07 | 0.68 | ±0.07 | 0.3784^‡^ |
| Total protein (mg/dL) | 6.77 | ±0.27 | 6.81 | ±0.32 |  | 6.67 | ±0.28 | 6.70 | ±0.31 | 0.7154^†^ |
| Albumin (g/dL) | 4.41 | ±0.18 | 4.49 | ±0.24 |  | 4.41 | ±0.16 | 4.48 | ±0.20 | 0.8648^†^ |
| Total bilirubin mg/dL) | 0.87 | ±0.27 | 0.81 | ±0.23 |  | 0.85 | ±0.22 | 0.72 | ±0.22 | 0.3032^‡^ |
| AST (IU/L) | 22.30 | ±3.82 | 20.67 | ±4.04 |  | 24.06 | ±6.58 | 22.60 | ±6.83 | 0.7164^†^ |
| ALT (IU/L) | 17.30 | ±5.29 | 14.60 | ±4.73 |  | 20.25 | ±11.82 | 16.79 | ±11.13 | 0.3959^‡^ |
| г-GT (U/L) | 8.75 | ±8.32 | 9.42 | ±14.74 |  | 12.38 | ±13.08 | 9.50 | ±7.70 | 0.0253^‡^ |
| HDL-C (mg/dL) | 57.15 | ±12.84 | 60.65 | ±13.75 |  | 62.06 | ±12.41 | 64.21 | ±13.95 | 0.5494^†^ |
| LDL-C (mg/dL) | 120.77 | ±26.43 | 117.94 | ±25.29 |  | 121.87 | ±30.33 | 125.13 | ±32.52 | 0.5434^‡^ |
| Triglyceride (mg/dL) | 98.09 | ±64.24 | 89.50 | ±41.69 |  | 101.50 | ±52.47 | 100.00 | ±57.04 | 0.8345^‡^ |
| Total-Cholesterol (mg/dL) | 191.53 | ±29.15 | 189.00 | ±25.79 |  | 200.75 | ±31.92 | 202.60 | ±31.14 | 0.7421^†^ |

^1^Comparison between groups, ^†^two sample t-test, ^‡^Wilcoxon rank sum test

**Supplementary Table 8**. Abbreviations and Glossary of Terms

| ADR | Adverse Drug Reaction |
| --- | --- |
| AE | Adverse Event |
| ALP | Alkaline Phosphatase |
| ALT | Alanine Aminotransferase |
| ANCOVA | Analysis of Covariance |
| AST | Aspartate Aminotransferase |
| EBN | Edible Bird's Nest |
| ESR | Erythrocyte Sedimentation Rate |
| BUN | Blood Urea Nitrogen |
| Corneometer | Skin moisture metre |
| CRP | C-reactive protein |
| Cutometer | Skin elasticity metre |
| FAS | Full Analysis Set |
| γ-GT | Gamma-glutamyltransferase |
| HDL-C | High-density lipoprotein cholesterol |
| HPLC | High Performance Liquid Chromatography |
| LDL-C | Low-density lipoprotein cholesterol |
| RnBS | Research & BioStatistics |
| R.H. | Relative humidity |
| GPDS | Global Photo Damage Score |
| IRB | Institutional Review Board |
| PPS | Per Protocol Set |
| RBC | Red Blood Cell |
| SV700 | Fine wrinkle analysis device |
| UNL | The Upper Normal Limit |
| WBC | White Blood Cell |
